# Push–Pull Zinc Phthalocyanine Bearing Hexa-Tertiary Substituted Carbazolyl Donor Groups for Dye-Sensitized Solar Cells

**DOI:** 10.3390/molecules25071692

**Published:** 2020-04-07

**Authors:** Basma Ghazal, Kobra Azizi, Ewies F. Ewies, Ahmed S. A. Youssef, Valid Mwatati Mwalukuku, Renaud Demadrille, Tomás Torres, Saad Makhseed

**Affiliations:** 1 Department of Chemistry, Kuwait University, P.O. Box 5969, Safat 13060, Kuwait; basmaghazal@gmail.com; 2 Organometallic and Organometalloid Chemistry Department, National Research Centre, Cairo 12622, Egypt; ef.ewies@nrc.sci.eg; 3 Departamento de Química Orgánica, Universidad Autónoma de Madrid, Cantoblanco, 28049 Madrid, Spain; azizikobra@ymail.com; 4 Department of Chemistry, Faculty of Science, Ain Shams University, Abbassia, P.O. 11566, Cairo, Egypt; ahmedy84@hotmail.com; 5 CEA-Univ. Grenoble Alpes-CNRS, IRIG, SyMMES, 38000 Grenoble, France; valid-mwatati.mwalukuku@cea.fr; 6 IMDEA-Nanociencia, Campus de Cantoblanco, 28049 Madrid, Spain

**Keywords:** Zn(II) phthalocyanine, A_3_B, dye-sensitized solar cells, DSSC

## Abstract

An asymmetrical, push–pull phthalocyanine bearing bulky *tert*-butylcarbazolyl moieties as electron donor and carboxylic acid as anchoring group was synthetized and tested as a photosensitizer in dye-sensitized solar cells (DSSC). The new photosensitizer was characterized by 1H and 13C NMR, UV–Vis and mass spectrometry. The bulky *tert*-butylcarbazolyl moieties avoid the aggregation of the phthalocyanine dye. DFT studies indicate that the HOMO is delocalized throughout the π-electron system of the substituted phthalocyanine and the LUMO is located on the core of the molecule with a sizable electron density distribution on carboxyl groups. The new dye has been used as a photosensitizer in transparent and opaque dye-sensitized solar cells, which exhibit poor efficiencies related to a low Jsc.

## 1. Introduction

Dye-sensitized solar cells (DSSCs) are an effective alternative for delivering clean energy from the sun compared to traditional power cells [1,2,3,4]. Numerous efforts are currently being directed toward the development of optimized light harvesters capable of utilizing the entire range of the solar spectrum, including the infrared portion lost in present silicon-based PV technology. These efforts have the potential to improve the efficiency in solar energy conversion of dye-sensitized solar cells (DSSCs) via the so-called panchromatic sensitization.

In a DSSC, the photosensitizer is a crucial element not only because it is responsible for the photon harvesting of the solar cell, but also because it drives the electron transfer at the TiO_2_/dye/electrolyte interfaces [5]. For this reason, it is of tremendous importance to accurately control not only the absorption but also the energy levels of the dyes. The structure engineering of organic dyes offers this possibility, and organic photosensitizers usually show superior absorption compared to ruthenium dyes. For this reason, a plethora of organic dyes have been developed in the last decade, leading to the improvement of the device performances and robustness [6,7].

The new generation of DSSCs use donor–π–acceptor (D–π–A) sensitizers with high molar absorptivity, which allow the use of TiO_2_ thin films, leading to improvements in the open circuit voltage V_OC_ when used with cobalt–based or copper–based redox mediators [1,8,9,10]. The production of efficient D–π–A sensitizers involves the design of panchromatic dyes with high absorption in the red and near-infrared regions as well as low aggregation [11]. Also, the ability of such dyes to establish strong electronic communication between the lowest unoccupied molecular orbitals (LUMOs) of the dye and the TiO_2_ conduction band ensures efficient electron injection from the excited photosensitizer into the semiconductor. Phthalocyanines (Pcs) represent one of the most prospective class of functional materials incorporated in DSSCs, owing to their high photochemical and electrochemical stabilities and extremely intense absorption band in the red-NIR region (i.e., the maximum solar flux of photons within this optical window region) [12,13]. Moreover, a strong light harvesting capability and high absorption at λ = 600–800 nm is the most photon-rich part of the sunlight.

The most successful ZnPcs sensitizers identified to date are asymmetrically substituted molecules of A_3_B type, characterized by three of the four isoindole subunits (called A), that makes up the Pc macrocycle as identical and bulky with electron-donating groups, whereas one or more anchoring groups (called B) are capable of interacting with the metal oxide semiconductor [14,15]. The aim of this A_3_B design is to combine steric suppression of dye aggregation, favorable electronic push–pull effects, and optimized binding mode of the dye with the semiconductor layer surface. Nazeeruddin, Torres et al., proposed this initially, using peripherally A_3_B form-bearing tert-butyl donors and -COOH anchoring groups that were able to achieve power conversion efficiencies up to 3.5% [15]. Molecular engineering toward Pc-based efficient DSSCs reported by Giribabu et al. involved substitution of the A_3_B ZnPcs with six butyloxy groups at the alpha peripheral positions and double carboxyl structures. Contrary to what was expected, the designed structures led to lower efficiency of 1.13% as the result of a low LUMO level, unsuitable for efficient electron injection [16,17]. Kimura et al. designed bulky, electron rich aryl substituents rotated with respect to the macrocycle plane. Even though the aggregation was strongly reduced, they reached the optimum PCE of 1.59% [18]. On the other hand, Torres, Nazeeruddin, Mori, Kimura et al. demonstrated the interest of a A_3_B ZnPc family, with electron-rich 2,6-disubstituted phenoxy groups, as a donor A, and one electron withdrawing -COOH group, with or without a rigid alkyne spacer between the dye and the anchoring group, as the acceptor B, reporting impressive power conversion efficiencies up to 6.1% (TT40) [19,20] and 6.4% (PcS20) [21,22,23] under simulated one-sun illumination. Regardless of continuous improvements in this field, the full potential of ZnPcs as a photosensitizer is yet to be realized in DSSCs and further developments are required.

Amino groups including NH_2_, NHR, -NR_2_, NHAr, and NAr_2_ have been tested as good donor groups to enhance the photovoltaic performance of asymmetrical ZnPcs (A_3_B) based on their higher LUMO levels, lower energy gaps, and red-shifted absorption bands [24,25]. Moreover, the presence of new absorption bands in the 400–600 nm region, where the molar absorption coefficient of the macrocycle is minimum, should make them good candidates as panchromatic sensitizers. Along the same lines are rational molecular designs by Giribabu et al. who prepared asymmetrical A_3_B Pc bearing three bulky electron-donating triphenylamines and two carboxyl units [24]. Regardless of the adequate optical design of the sensitizer, it exhibited poor performance. Meanwhile, the IPCE (Incident Photon-to-electron Conversion Efficiency) spectra showed broader, red-shifted, and better response [1,24]. Several attempts have been reported to enhance the overall power conversion efficiency PCE along with adequate optical properties [25,26]. Using a different approach, Kimura et al. described the direct annulation of carbazole entities with the Pc core and revealed an influence of carbazole groups on the photophysical and electrochemical properties as well as on their performance in DSSC [27], in addition to interesting wide light-harvesting properties in the 400–800 nm range. Recently, we reported an octa-substituted carbazolyl phthalocyanines with a broad absorption band observed in the 450–500 nm range [28,29]. The steric hindrance imposed by bulky *tert*-butyl groups on the carbazole substituents are expected to suppress the undesirable molecular aggregation while at the same time acting as electron-donating units. This should provide excellent absorption in the NIR region.

Herein, we have designed and synthesized an asymmetrical phthalocyanine (**tCzZnPc1**) bearing six *tert*-butylcarbazolyl moieties and one carboxyl group, and this new molecule was incorporated for the first time in DSSCs as a photosensitizer.

## 2. Results and Discussion

### 2.1. Synthesis

**Scheme 1:**
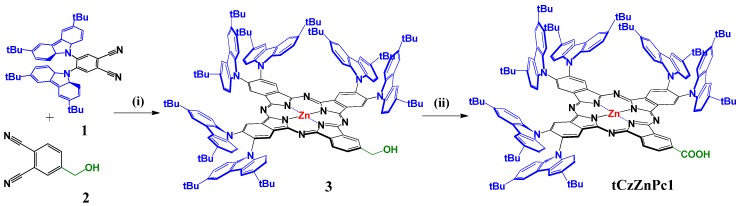
Synthetic route to peripherally substituted A_3_B **tCz-ZnPc** with carboxylic anchoring group (i) Zn(AcO)_2_, DMAE, reflux (ii) ZnO, KOH, mesitylene, reflux.

The synthetic route to **tCzZnPc1** is depicted in Scheme 1. The molecule has been prepared according to Zn(II)-templated cyclization on a mixture of 4,5-bis(3,6-di-tert-butyl-9H-carbazol-9-yl)phthalonitrile (**1**) and 4-(hydroxymethyl)phthalonitrile (**2**) as building blocks, (molar ratio 4:1) to afford the A_3_B ZnPc (**3**) in 14% yield. The dehydrogenation of hydroxymethyl phthalocyanine 3 was performed in zinc oxide and potassium hydroxide solution in mesitylene to give **tCzZnPc1** in 92% overall yield and hydrogen gas was the only byproduct (for more details, see the Materials and Methods section). The catalytically active species in the solution is believed to be the corresponding zinc alkoxide from the reaction of alcohol with zinc oxide and potassium hydroxide. Decomposition of the alkoxide would result in zinc hydride and the corresponding aldehyde, which can give rise to the carboxylate and the starting alcohol by either a Cannizzaro reaction or a Tishchenko reaction in the presence of KOH [30].

**Figure 1:**
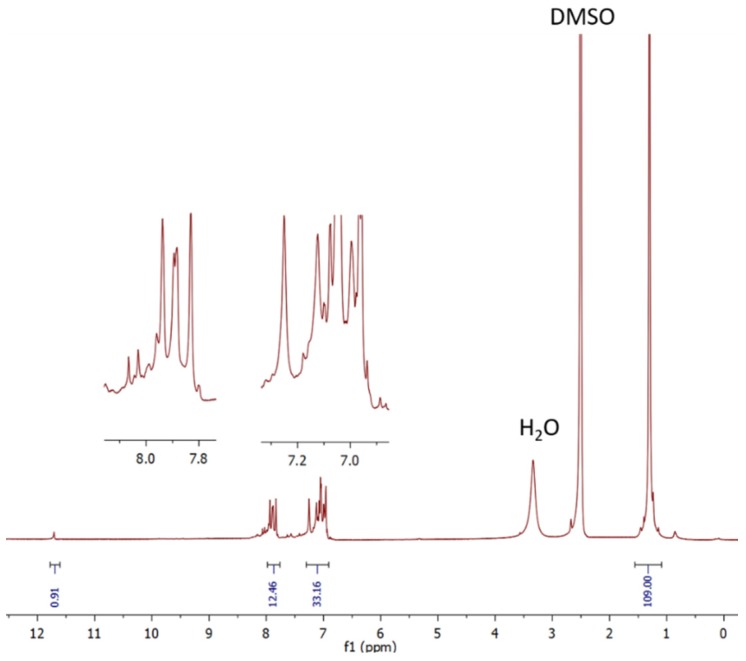
^1^H NMR spectrum for A_3_B **tCzZnPc1** (in DMSO-*d*_6_).

Both **3** and **tCzZnPc1** complexes are freely soluble in most organic solvents. Structural identification of both was achieved by ^1^H (Figure 1), and ^13^C NMR spectroscopies as well as UV-Vis and MALDI-TOF spectrometry. The UV-Vis spectra of **3** (Figure S4) and **tCzZnPc1** were detected in THF and exhibited the classic Q-band of phthalocyanines, and typical shape of monomeric systems. A red-shift of this Q-band, regarding common substituted phthalocyanines, at around 707 nm was observed. The intensity of this absorption in both **3** and **tCzZnPc1** is low, particularly in the last one. A strong absorption around ~340 nm, due to the carbazole moieties, appeared overlapping in the B-band region in **tCzZnPc1** (Figure 4a), which was observed before in a previously prepared symmetrical octacarbazolylphthalocyanine Pc [25]. In our particular case, the intensity of the Q-band in **tCzZnPc1** is low in comparison with the strong absorption of the six carbazole moieties which absorb in the region of 340 nm. On the other hand, it is well known that phthalocyanines bearing bulky groups can be highly distorted, with deviation of the planarity, which can affect the intensity of this band.

**Figure 2:**
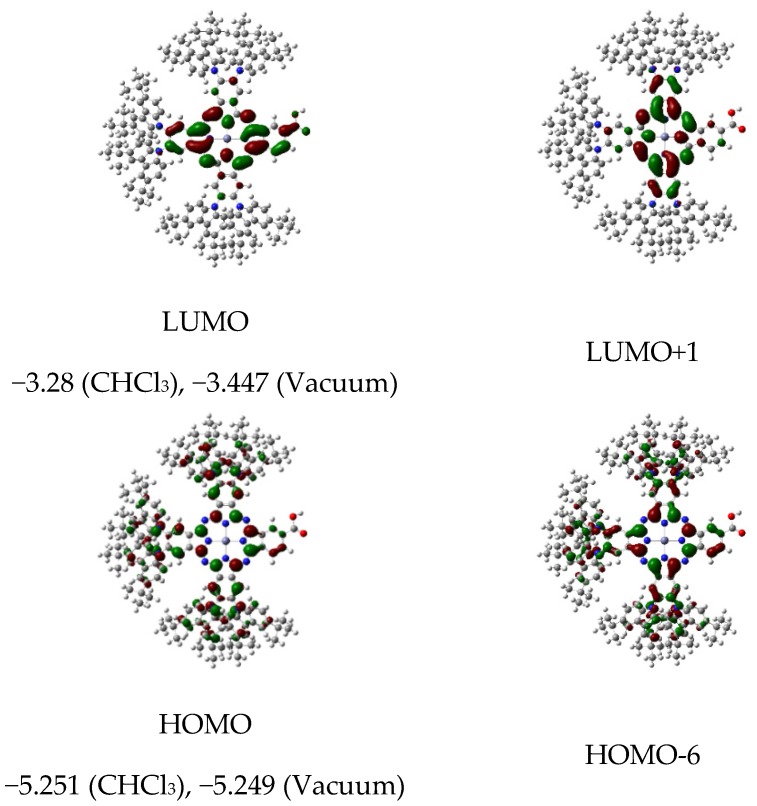
The Gouterman’s molecular orbitals of **tCzZnPc1** calculated at CAM-B3LYP/LANL2DZ level of theory, and the calculated values of HOMO and LUMO energy levels in a vacuum and in CHCl_3_.

Having proved the synthetic feasibility of A_3_B **tCzZnPc1** substituted with six tert-butylcarbazolyl moieties, we then examined the electronic compatibility of **tCzZnPc1** for use as a sensitizer in a DSSC configuration. The architecture of a DSSC requires the favorable energy alignment of all functional components of the electrochemical circuit, such as the electron transport material (ETM), the sensitizer, and the redox couple responsible for the regeneration of the photo-oxidized dye. Theoretical calculations were carried out to understand the geometrical configuration, electronic distributions, and frontier orbital energies of **tCzZnPc1** using TD−DFT on CAM-B3LYP exchange–correlation functional [31] coupled with the double-zeta quality LANL2DZ basis set [32] for all atoms. **tCzZnPc1** reveals good spatial orbitals separation. The HOMO levels of the dye are delocalized over pyrrolic α- and β-pyrrolic carbons in the studied complexes along with high contribution of carbazole donor moieties. Meanwhile, the LUMO levels are delocalized at the core, with contributions from either the α- and β-pyrrolic carbon atoms, or from meso- and inner-nitrogen atoms along with a sizable electron density distribution on carboxylic anchoring group (Figure 2).

### 2.2. Photovoltaic Performance

**Figure 3:**
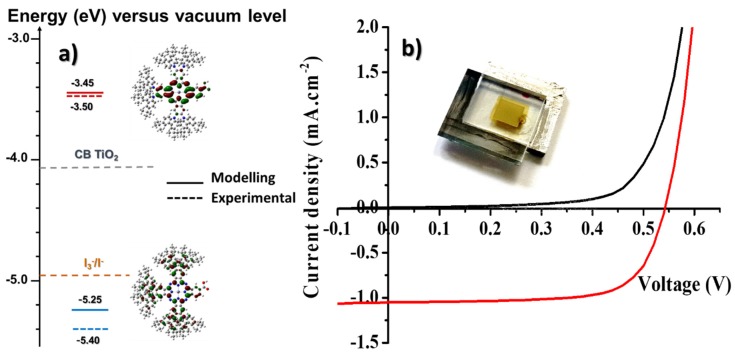
(**a**) Energy band diagram of the DSSC fabricated in this work. (**b**) Current–voltage curves of an opaque **tCzZnPc1**-based device recorded in the dark (black line) and under irradiation (red line) under AM 1.5G (1000 W·m^−2^) irradiation condition.

In order to evaluate the potential of the newly synthesized molecules for photovoltaic applications, semi-transparent and opaque DSSCs were fabricated following a standard procedure. The opaque solar cells refer to devices that include an additional TiO_2_ layer (particle size ~ 400 nm) of about 3 to 4 µm thick above the mesoporous TiO_2_, whereas the “transparent solar cells” contain only the mesoporous TiO_2_ layer of 13 µm-thick layer (particle size ~ 15–20 nm). The devices show an active area of 0.36 cm^2^. The complete treatment of the electrodes are fully described in the experimental section. Immediately after the treatment, the photoanodes were sensitized through immersion in the dyeing solution for 16 h at room temperature with varying dye concentration of **tCzZnPc1** = 0.2 and 0.5 mM, with/without CDCA co-adsorption due to the bulkiness of our dye, in CHCl_3_/EtOH 1/1, *v*/*v*. The CDCA coadsorption was done with the dye/CDCA ratio of 1:5. Platinized counter electrodes were employed in this study and the cells were filled with an electrolyte (Solaronix Iodolyte HI-30) via the pre-drilled hole using a vacuum pump. To improve the current collection, a metal contact along the cell edges was deposited using an ultrasonic solder. Before measurements, the AM1.5G solar simulator was calibrated using a reference silicon photodiode equipped with an IR-cutoff filter (KG-3, Schott). The current–voltage characteristics of the cells were measured under dark and under AM 1.5G (1000 W·m^−2^) irradiation condition. The devices were masked prior to measurements to attain an illuminated active area of 0.36 cm^2^ (Figure 3).

From the HOMO and LUMO energy levels determined by cyclic voltammetry in solution, it is clear that the molecule can be used as a photosensitizer in a classical DSSC configuration. Experimental HOMO and LUMO energy levels are found in relatively good agreement with the results from the modeling. We determined a HOMO energy level located at -5.40 eV and a LUMO level located at −3.50 eV. Taking into account that the conduction band energy level of the semiconductor is around −4.1 eV and the redox potential of the I^−^/I_3_^−^ is around −3.9 eV, the driving force for the electron photoinjection is higher than 0.5 eV, and the driving force for the regeneration of the dye is higher than 0.3 eV.

Despite this, the photovoltaic parameters of the solar cells fabricated in this work were quite disappointing (see Table 1). One should note that the electrodes are yellowish after sensitization, showing that the absorption of the dye overlaps strongly with the one of the iodine-based electrolytes. The highest performance was achieved with an opaque device. The best cell exhibited a Jsc of 1.05 mA.cm^−2^, Voc of 542 mV, and FF of 70.8%, leading to a power conversion efficiency of 0.40%. Surprisingly, the transparent solar cells showed a quite similar PCE around 0.35% with a Jsc of 0.91 mA.cm^−2^, Voc of 538 mV, and FF of 71.7%. These results were reproducible over three devices. The use of chenodeoxycholic, which is often employed to reduce aggregation of the dyes on TiO_2_ surface, did not improve the performances of the cells. This result was expected because of the presence of the twelve tertbutyl groups on the molecule that are supposed to totally suppress aggregation phenomenon. Unfortunately, the very poor performances and the low Jsc impede accurate IPCE analysis of the solar cells.

**Figure 4:**
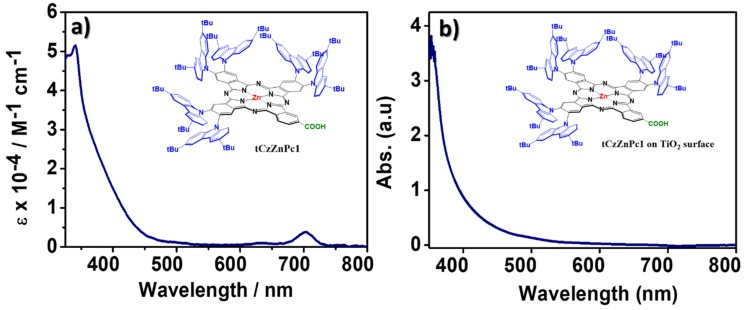
(**a**) Absorption in solution **tCzZnPc1** (THF, 25 °C, 10^−5^ M), and (**b**) after adsorption on a 4µm-thick transparent TiO_2_ mesoporous electrode.

In order to find the reason behind the poor efficiency of this new Zn-phthalocyanine derivative, we have measured the molar absorption coefficient in THF solution and the absorption spectrum of the molecule after adsorption on TiO_2_ mesoporous transparent electrodes (see Figure 4) and calculated the dye loading on the opaque photoelectrodes.

These measurements demonstrate that the molecule has poor absorption in the visible range. After grafting the molecule on TiO_2_, the absorption spectrum is slightly enlarged with an absorption edge close to 600 nm, but the absorption feature corresponding to the Q-band disappears. In addition, a very low dye-loading of 6.069 × 10^−8^ moles·cm^−2^ was estimated on the 13 + 4 µm-thick photoelectrodes. These results can explain the low Jsc values and consequently low performance of the molecule. Our preliminary results on this class of molecules indicate that the introduction of bulky substituents is a good strategy to reduce the dye aggregation but their low absorption in the visible range and the low amount of dyes attached on the surface lead to poor photovoltaic performances.

## 3. Materials and Methods

### 3.1. General Methods and Characterization Techniques

Carbazole, deuterated dimethylsulfoxide (DMSO-d_6_), cesium fluoride (CsF), anhydrous zinc acetate, ZnO, and KOH were purchased from Sigma-Aldrich. 5,6-Dichlorophthalonitrile was obtained from TCI (Toshima, Japan). Mesitylene (solvent) from Sigma-Aldrich was stored over activated 4Å molecular sieves (HPLC grade) and degassed with N_2_-vacuum before use. Anhydrous solvents were either supplied from Sigma-Aldrich and used as they were received and dried with 4Å molecular sieves (Panreac) or dried as described by Perrin [33]. TLC was performed using polygram sil G/UV 254 (0.2 mm thick, E. Merck, Darmstadt, Germany) TLC plates and visualization was carried out by ultraviolet light at 254 and 350 nm. Column chromatography was performed using Merck silica gel 60, 230–400 of mesh size 0.040–0.063 mm, Merck and eluents are indicated for each particular case. Gel permeation chromatography (GPC) was performed using Bio-Beads S-X1 (200400 mesh, Bio-Rad, Hercules, CA, USA). NMR spectra were recorded on a Bruker Ascend 400 spectrometer.

^1^H and ^13^C NMR spectra were recorded using Bruker DPX 600 at 600 and 150 MHz, respectively, while for oxidized complex, the NMR spectra were recorded on a Bruker Ascend 400 spectrometer. The IR spectra were obtained using Jasco 6300 FTIR. UV–Vis spectra were recorded with a Varian Cary 5 or Shimadzu UV-2600 spectrophotometer. MALDI-TOF MS was performed on a Bruker ultrafleXtreme using RP 700-3500-Da LP-700-3500-Da methods and 2, 5-dihydroxybenzoic acid (DHB) as the matrix on a ground steel plate. The MALDI-TOF mass data for Pcs are presented as the mass of the most intense peak in the cluster instead of exact mass. LC–MS was performed on a Waters ACQUITY UPLC system equipped with PDA and SQD2 electrospray MS detector. The parameters of operation were column: Thermo Accucore C18 (2.6 µm, 2.1 × 50 mm); column temp: 50 °C; flowrate: 0.6 mL/min;solvent A: 5mM NH4Ac in water; solvent B: 5mM NH4Ac in acetonitrile/water (95:5).

### 3.2. Computational Details

The starting geometries of Pz **tCzZnPc1** were optimized using CAM-B3LYP/LANL2DZ [31,32] for all atoms. Frequency calculation was used to confirm the energy minima in optimized geometry. Chloroform was used as a solvent in all of the single point DFT–PCM and TDDFT–DCM calculations were performed using the polarizable continuum model (PCM) to study the solvation effects [34]. The first 100 excited states of each compound were calculated in all TDDFT–PCM calculations. All DFT calculations were conducted using the Gaussian 09 software package [35], and the GaussView program [36] was used for the molecular orbital analysis.

### 3.3. Synthesis

Compounds 4,5-bis(3,6-di-tert-butyl-9H-carbazol-9-yl)phthalonitrile (**1**) and 4-(hydroxymethyl)phthalonitrile (**2**), [37] have been prepared according to published procedures.

*Tri-tert-butylcarbazolyl-hydroxymethylphthalocyaninatozinc(II)* (**3**)*:* A mixture of 4,5-bis(3,6-di-tert-butyl-9H-carbazol-9-yl)phthalonitrile (**1**) (737 mg, 4 mmol), 4-hydroxymethylphthalonitrile (**2**) [37] (158 mg, 1 mmol) and Zn(AcO)_2_ (330 mg, 1.5 mmol) in DMAE (4 mL) was refluxed during 24 h. The solvent was evaporated under reduced pressure, and the crude product was purified by column chromatography (SiO2, ethyl acetate/cyclohexane 9:1) to afford **3** as a deep green solid (130 mg, 14%); m.p. > 300 °C; ^1^H NMR (600 MHz, DMSO-*d*_6_) δ = 9.82 (d, 6H), 9.82 (s, 1H), 9.43 (d, 2H), 8.58 (s, 1H), 7.96–7.79 (m, 12H), 7.39–7.30 (m, 12H), 7.13–7.05 (m, 12H), 6.07 (bs, 3H), 1.37–1.30 (brs, 108H). ^1^H NMR (600 MHz, benzene-*d*_6_) δ = 10.37 (s, 1H), 10.25–10.18 (b, 6H), 9.46 (bs, 2H), 7.98–7.84 (m, 12H), 7.45–7.06 (m, 24), 4.69 (s, 3H), 0.91-0.29 (brs, 108H). ^13^C NMR (100 MHz, DMSO-*d*_6_) δ = 168.4, 143.3, 139.1, 138.2, 137.8, 132.8, 127.4, 124.0, 123.8, 123.4, 123.2, 109.5, 31.7, 31.6 ppm. UV-Vis (THF) *λ*_max_ (nm) (log *ε* (dm^3^ mol^−1^ cm^−1^)): 707 (5.5), 350 (5.1). MS (MALDI-TOF, DHB on ground steel plate): *m*/*z*: 2272.56.

*Tri-tert-butylcarbazolyl-carboxylphthalocyaninatozinc(II)* (**tCzZnPc1**)*:* Zinc oxide (0.4 mg, 0.004 mmol) and potassium hydroxide (2.8 mg, 0,04 mmol) were placed in an oven-dried tube, which was placed in a Radley carousel. The tube was subjected to vacuum and then filled with nitrogen gas (repeated three times). Vacuum was applied again, and the carousel heated to 170 °C for 1 h. Subsequently, the tube was refilled with nitrogen gas. Anhydrous and degassed mesitylene (2 mL) was added and the mixture heated to reflux. **3** (0.02 mmol, 40 mg) that previously was dissolved in degassed mesitylene (1 mL) was added dropwise by syringe, and the reaction was refluxed with stirring under a flow of nitrogen for 24 h. The mixture was cooled down to room temperature and the mesitylene was evaporated under vacuum. The precipitate was acidified with 16% aqueous hydrochloric acid. The aqueous layer was extracted with ethyl acetate (3 × 5 mL). The combined organic layers were dried over sodium sulfate and concentrated in vacuum to give **tCzZnPc1** as a pure compound (36 mg, 92%). m.p. > 300 °C; ^1^H NMR (400 MHz, DMSO-*d*_6_) δ = 11.72 (s, 1H), 7.96–7.76 (m, 12H), 7.32–6.92 (m, 33H), 1.30 (brs, 109H). ^13^C NMR (100 MHz, DMSO-*d*6) δ = 168.4, 143.3, 139.1, 138.2, 137.8, 132.8, 127.4, 124.0, 123.8, 123.4, 123.2, 109.5, 34.7, 32.1 ppm. UV-Vis (THF) *λ*_max_ (nm) (log ε(dm^3^ mol^−1^ cm^−1^)): 707 (3.7), 355 (4.5 sh). MS (MALDI-TOF, DHB on ground steel plate): *m*/*z*: 2285.74.

### 3.4. Photovoltaics

The devices reported in this paper were prepared using the following procedure. TiO_2_ thin films with specific thickness and a total area of 0.36 cm^2^ were screen printed in Solaronix (Switzerland) using a TiO_2_ nanoparticles paste (Ti-nanoxide HT/SP). The “opaque solar cells” are based on a 13µm-thick mesoporous TiO_2_ layer with an additional 4 µm-thick scattering (Solaronix, Ti-Nanoxide R/SP) whereas the “transparent solar cells” contain only the 13 µm-thick mesoporous TiO_2_ layer. The electrodes are cleaned with absolute ethanol beforehand and dried under an argon flow. The photoanodes are then treated by immersion into a freshly prepared 4.1 mmol·L^−1^ TiO_2_ aqueous suspension at 70 °C for 20 min. The electrodes are then cooled down to room temperature, rinsed with distilled water followed by absolute ethanol, and then drying under an argon flux. The electrodes are then sintered under air at 500 °C for 20 min. The photoanodes are then cooled down to 80 °C and sensitized through immersion in the dyeing solution for 16 h at room temperature with varying dye concentration of **tCzZnPc1** = 0.2 and 0.5 mM, with/without CDCA co-adsorption, due to the bulkiness of our dye, in CHCl3/EtOH 1/1, *v*/*v*. The CDCA coadsorption was done with the dye: CDCA ratio of 1:5. The drilled counter electrodes are coated with a thin layer of platisol (Solaronix, Switzerland) and charred under air at 500 °C using the same heating procedure as described above. The sensitized photoanode is rinsed with dichloromethane, absolute ethanol, and dried under an argon flux. Both electrodes are then sandwiched together using a suryln thermogluing polymer (60 µm thick) using a heating press at 105 °C for 16 s. The cell is then filled with an electrolyte (Solaronix Iodolyte HI-30) via the pre-drilled hole using a vacuum pump. The electrolyte injection hole on the counter electrode is then sealed with the aid of a surlyn underneath the thin glass cover using heat. Lastly, contact along the cell edges is created.

Before measurements, the AM1.5G simulator was calibrated using a reference silicon photodiode equipped with an IR-cutoff filter (KG-3, Schott). The current–voltage characteristics of the cells were measured under dark and under AM 1.5G (1000 W m^−2^) irradiation condition, achieved by applying an external potential bias to the cell while measuring the generated photocurrent with a Keithley model 2400 digital source meter (Beaverton, OR, USA). The devices were masked prior to measurements to attain an illuminated active area of 0.36 cm^2^.

## 4. Conclusions

An asymmetrical, push–pull phthalocyanine bearing six bulky tert-butylcarbazolyl electron donor units and a carboxylic acid anchoring group were synthetized and tested as photosensitizer in dye-sensitized solar cells (DSSC). DFT and electrochemical studies indicate that the electronic distribution in the dye is appropriate for electron injection into the titanium dioxide. The new dye has been used as photosensitizer in transparent and opaque dye-sensitized solar cells, which exhibit poor efficiencies related to a low Jsc. These preliminary results indicate that the introduction of bulky carbazolyl substituents is a good strategy to reduce the dye aggregation but its low absorption in the visible range and the low amount of dyes attached on the TiO_2_ surface may explain the poor photovoltaic parameters.

## Figures and Tables

**Table 1:** Photovoltaic parameters of the best solar cells and mean values over 3 cells (in parenthesis).

Dye	Cells	J_SC_ (mA·cm^−2^)	V_OC_ (V)	FF (%)	PCE (%)
**tCzZnPc1**	Opaque	1.05 (0.98)	0.54 (0.54)	70.8 (69.8)	0.40 (0.37)
**tCzZnPc1**	Transparent	0.91 (0.77)	0.54 (0.51)	71.7 (73.3)	0.35 (0.29)

## Supplementary Materials

The following are available online at https://www.mdpi.com/1420-3049/25/7/1692/s1, Mass spectra: Figures S1 and S2; NMR spectrum: Figure S3; Absorption and Emission spectra: Figures S4 to S13; Electrochemical data: Figures S14 to S18.

## References

[1] Urbani M., Ragoussi M.-E., Nazeeruddin M.K., Torres T. (2019). Phthalocyanines for dye-sensitized solar cells. Coord. Chem. Rev..

[2] O’Regan B., Grätzel M. (1991). A low-cost, high-efficiency solar cell based on dye-sensitized colloidal TiO_2_ films. Nature.

[3] Grätzel M. (2009). Recent advances in sensitized mesoscopic solar cells. Acc. Chem. Res..

[4] Ragoussi M.-E., Torres T. (2015). New generation solar cells: Concepts, trends and perspectives. Chem. Commun..

[5] Ooyama Y., Harima Y. (2012). Photophysical and electrochemical properties, and molecular structures of organic dyes for dye-sensitized solar cells. ChemPhysChem.

[6] Mishra A., Fischer M.K.R., Bäuerle P. (2009). Metal-free organic dyes for dye-sensitized solar cells: from structure: property relationships to design rules. Angew. Chem. Int. Ed..

[7] Wang P., Yang L., Wu H., Cao Y., Zhang J., Xu N., Chen S., Decoppet J.-D., Zakeeruddin S.M., Grätzel M. (2018). Stable and efficient organic dye-sensitized solar cell based on ionic liquid electrolyte. Joule.

[8] Feldt S.M., Wang G., Boschloo G., Hagfeldt A. (2011). Effects of driving forces for recombination and regeneration on the photovoltaic performance of dye-sensitized solar cells using cobalt polypyridine redox couples. J. Phys. Chem. C.

[9] Harrath K., Hussain Talib S., Boughdiri S. (2018). Theoretical design of metal-phthalocyanine dye-sensitized solar cells with improved efficiency. J. Mol. Model..

[10] Freitag M., Daniel Q., Pazoki M., Sveinbjörnsson K., Zhang J., Sun L., Hagfeldt A., Boschloo G. (2015). High-efficiency dye-sensitized solar cells with molecular copper phenanthroline as solid hole conductor. Energy Environ. Sci..

[11] Aumaitre C., Rodriguez-Seco C., Jover J., Bardagot O., Caffy F., Kervella Y., López N., Palomares E., Demadrille R. (2018). Visible and near-infrared organic photosensitizers comprising isoindigo derivatives as chromophores: Synthesis, optoelectronic properties and factors limiting their efficiency in dye solar cells. J. Mater. Chem. A.

[12] Bian Y., Chen J., Xu S., Zhu L., Zhou Y., Xiang Y., Xia D. (2015). Self-assembled core–shell nanospheres and dendritic nanostructure of novel tetra-(3-phenyprop-2-allyloxy) phthalocyanine in different solvents. RSC Adv..

[13] Lim J., Yeap S.P., Che H.X., Low S.C. (2013). Characterization of magnetic nanoparticle by dynamic light scattering. Nanoscale Res. Lett..

[14] Reddy P.Y., Giribabu L., Lyness C., Snaith H.J., Vijaykumar C., Chandrasekharam M., Lakshmikantam M., Yum J.-H., Kalyanasundaram K., Grätzel M. (2007). Efficient sensitization of nanocrystalline TiO_2_ films by a near-IR-absorbing unsymmetrical zinc phthalocyanine. Angew. Chem. Int. Ed..

[15] Cid J.-J., Yum J.-H., Jang S.-R., Nazeeruddin M.K., Martínez-Ferrero E., Palomares E., Ko J., Grätzel M., Torres T. (2007). Molecular cosensitization for efficient panchromatic dye-sensitized solar cells. Angew. Chem. Int. Ed..

[16] Giribabu L., Vijay Kumar C., Gopal Reddy V., Yella Reddy P., Srinivasa Rao C., Jang S.-R., Yum J.-H., Nazeeruddin M.K., Grätzel M. (2007). Unsymmetrical alkoxy zinc phthalocyanine for sensitization of nanocrystalline TiO_2_ films. Solar Energy Mater. Solar Cells.

[17] Giribabu L., Kumar C.V., Reddy P.Y., Yum J.-H., Grätzel M., Nazeeruddin M.K. (2009). Unsymmetrical extended π-conjugated zinc phthalocyanine for sensitization of nanocrystalline TiO_2_ films. J. Chem. Sci..

[18] Nagata M., Kimura M., Taya M. (2008). Design of Dye-Sensitized Solar Cells with New Light-Harvesting Dyes.

[19] Ragoussi M.-E., Cid J.-J., Yum J.-H., de la Torre G., Di Censo D., Grätzel M., Nazeeruddin M.K., Torres T. (2012). Carboxyethynyl anchoring ligands: a means to improving the efficiency of phthalocyanine-sensitized solar cells. Angew. Chem. Int. Ed..

[20] Ragoussi M.-E., Yum J.-H., Chandiran A.K., Ince M., de la Torre G., Grätzel M., Nazeeruddin M.K., Torres T. (2014). Sterically hindered phthalocyanines for dye-sensitized solar cells: influence of the distance between the aromatic core and the anchoring group. ChemPhysChem.

[21] Mori S., Nagata M., Nakahata Y., Yasuta K., Goto R., Kimura M., Taya M. (2010). Enhancement of incident photon-to-current conversion efficiency for phthalocyanine-sensitized solar cells by 3D molecular structuralization. J. Am. Chem. Soc..

[22] Matsuzaki H., Murakami T.N., Masaki N., Furube A., Kimura M., Mori S. (2014). Dye aggregation effect on interfacial electron-transfer dynamics in zinc phthalocyanine-sensitized solar cells. J. Phys. Chem. C.

[23] Ikeuchi T., Nomoto H., Masaki N., Griffith M.J., Mori S., Kimura M. (2014). Molecular engineering of zinc phthalocyanine sensitizers for efficient dye-sensitized solar cells. Chem. Commun..

[24] Giribabu L., Singh V.K., Kumar C.V., Soujanya Y., Reddy P.Y., Kantam M.L. (2011). Triphenylamine–phthalocyanine based sensitizer for sensitization of nanocrystalline TiO_2_ films. Solar Energy.

[25] Ince M., Cardinali F., Yum J.-H., Martínez-Díaz M.V., Nazeeruddin M.K., Grätzel M., Torres T. (2012). Convergent synthesis of near-infrared absorbing, “Push–Pull”, Bisthiophene-substituted, Zinc(II) phthalocyanines and their application in dye-sensitized solar cells. Chem. A Eur. J..

[26] Milan R., Selopal G.S., Cavazzini M., Orlandi S., Boaretto R., Caramori S., Concina I., Pozzi G. (2017). Dye-sensitized solar cells based on a push-pull zinc phthalocyanine bearing diphenylamine donor groups: Computational predictions face experimental reality. Sci. Rep..

[27] Kimura M., Suzuki H., Tohata Y., Ikeuchi T., Yamamoto S., Kobayashi N. (2017). Carbazole-fused Zinc(II)−phthalocyanine sensitizers. Asian J. Org. Chem..

[28] Majeed S.A., Ghazal B., Nevonen D.E., Goff P.C., Blank D.A., Nemykin V.N., Makhseed S. (2017). Evaluation of the intramolecular charge-transfer properties in solvatochromic and electrochromic Zinc Octa(carbazolyl)phthalocyanines. Inorg. Chem..

[29] Majeed S.A., Ghazal B., Nevonen D.E., Nemykin V.N., Makhseed S. (2019). Spectroscopic and TDDFT studies on the charge-transfer properties of metallated Octa(carbazolyl)phthalocyanines. Dye. Pigment..

[30] Monda F., Madsen R. (2018). Zinc oxide-catalyzed dehydrogenation of primary alcohols into carboxylic acids. Chem. A Eur. J..

[31] Yanai T., Tew D.P., Handy N.C. (2004). A new hybrid exchange–correlation functional using the Coulomb-attenuating method (CAM-B3LYP). Chem. Phys. Lett..

[32] Dunning T.H., Hay P.J. (1976). Modern Theoretical Chemistry.

[33] Kieboom A.P.G., Perrin D.D., Armarego W.L.F. (1988). Purification of Laboratory Chemicals.

[34] Tomasi J., Mennucci B., Cammi R. (2005). Quantum mechanical continuum solvation models. Chem. Rev..

[35] Frisch M.J., Trucks G.W., Schlegel H.B., Scuseria G.E., Robb M.A., Cheeseman J.R., Scalmani G., Barone V., Petersson G.A., Nakatsuji H. (2016). Gaussian 16.

[36] Dennington R., Keith T., Millam J. (2009). GaussView, Version 5.

[37] Enes R.F., Cid J.-J., Hausmann A., Trukhina O., Gouloumis A., Vázquez P., Cavaleiro J.A.S., Tomé A.C., Guldi D.M., Torres T. (2012). Synthesis and photophysical properties of fullerene–Phthalocyanine–Porphyrin triads and pentads. Chem. A Eur. J..

